# Supplementary material for: Dysregulations of Key Regulators of Angiogenesis and Inflammation in Abdominal Aortic Aneurysm
Source: Int J Mol Sci. 2023 Jul 28;24(15):12087. doi: 10.3390/ijms241512087 (PMC10418409; doi:10.3390/ijms241512087)
Supplement: Supplementary file 1 [file ijms-24-12087-s001.zip › Supplementary Figures.pdf]

SUPPLEMENTARY FIGURES

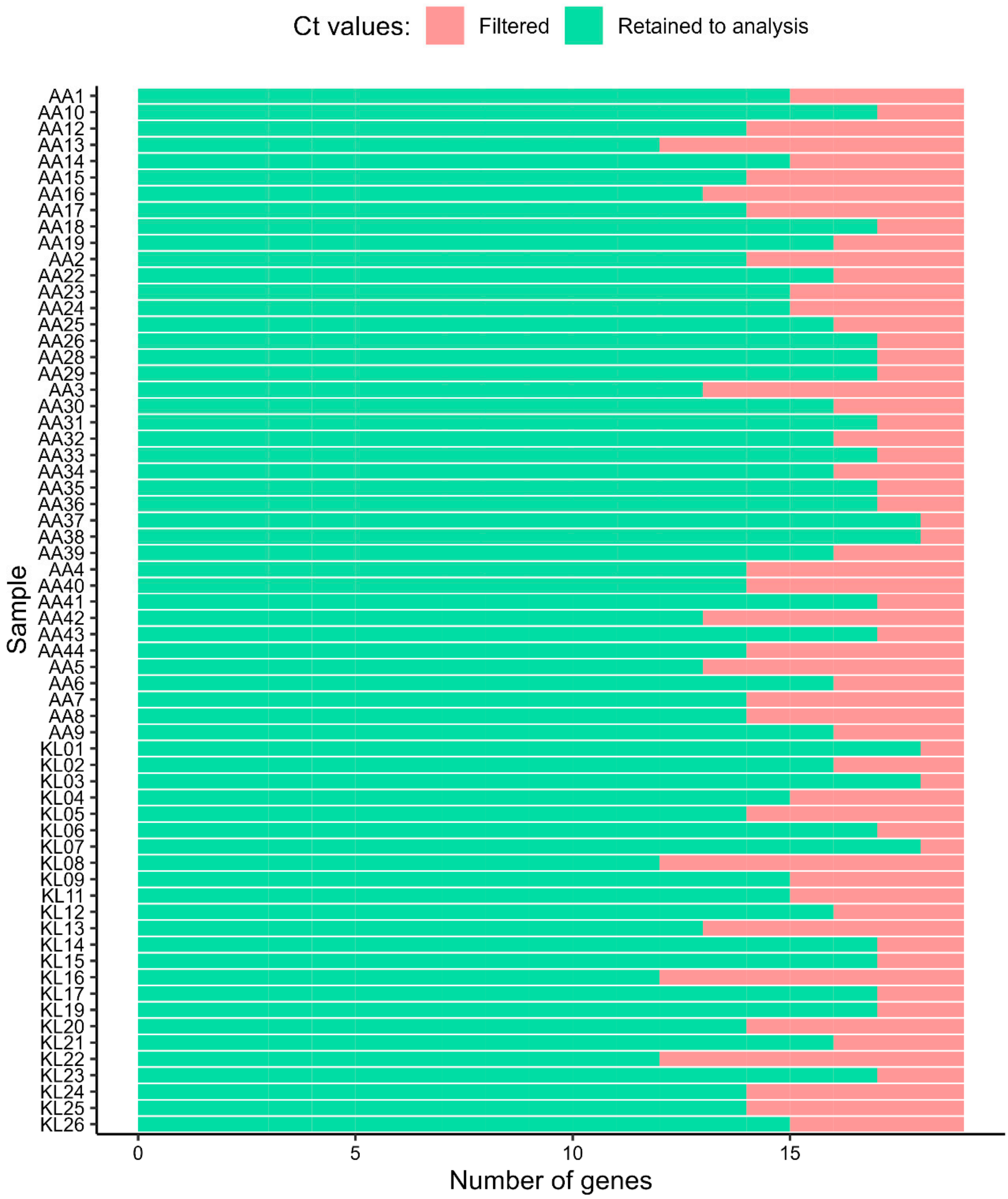

Figure S1. The amounts of genes with filtered and retained Ct values in analyzed samples.

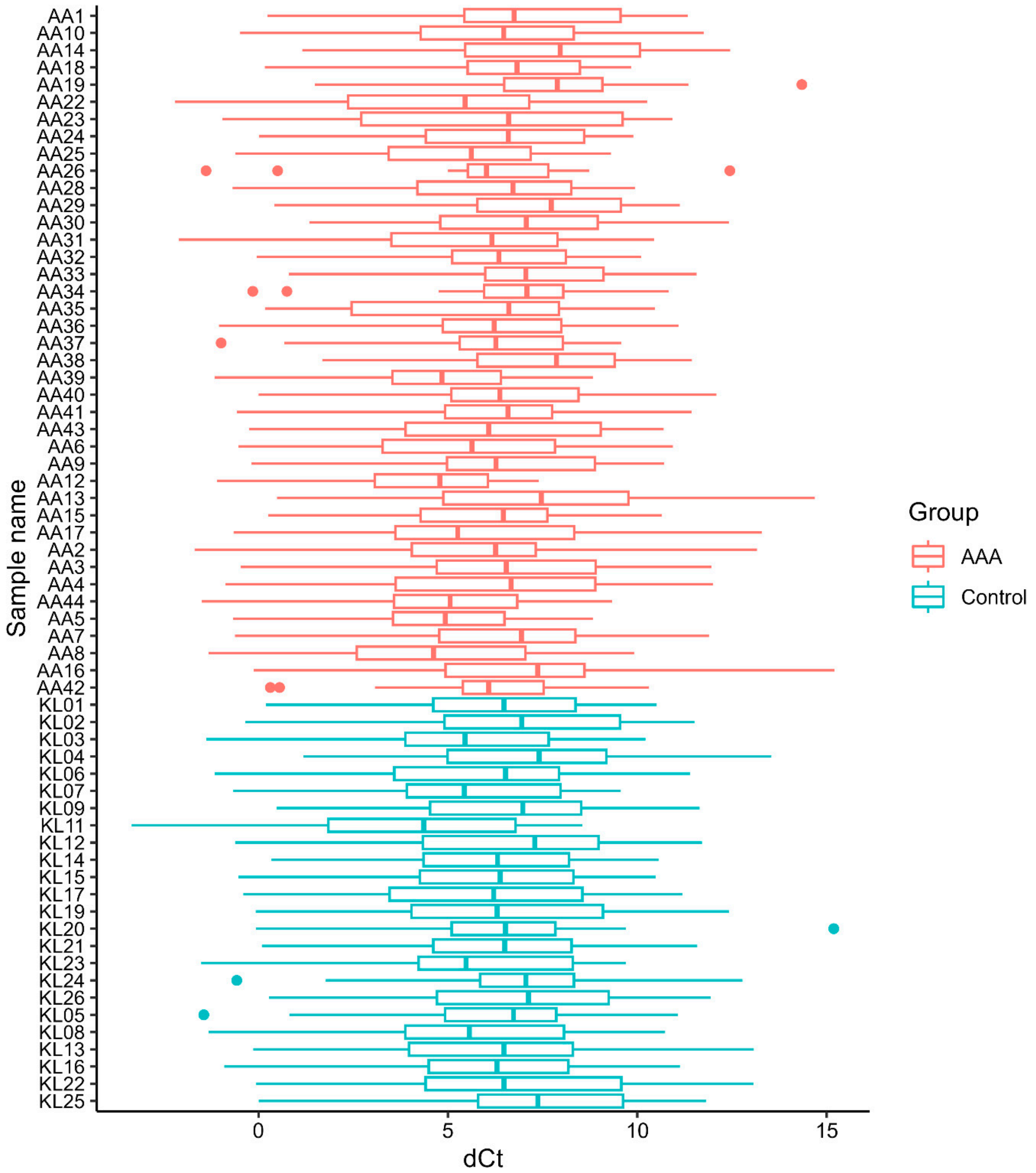

Figure S2. Distributions of dCt values in analyzed samples. Whiskers reach the most distant point in doubled interquartile range, boxes range between 25% and 75% quartile, horizontal lines inside boxes mark median value.

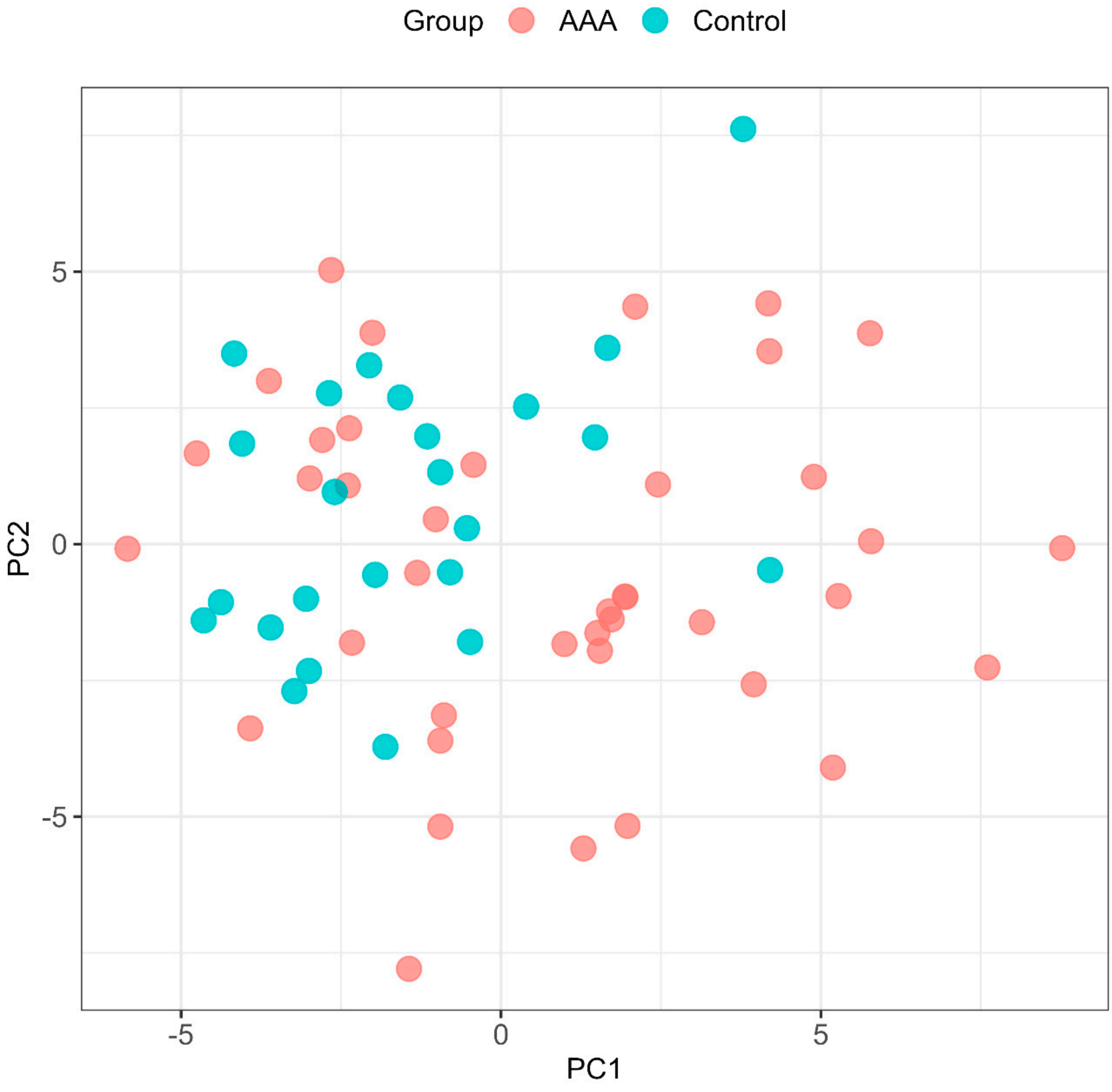

Figure S3. Spatial arrangement of samples using PCA components of dCt data.

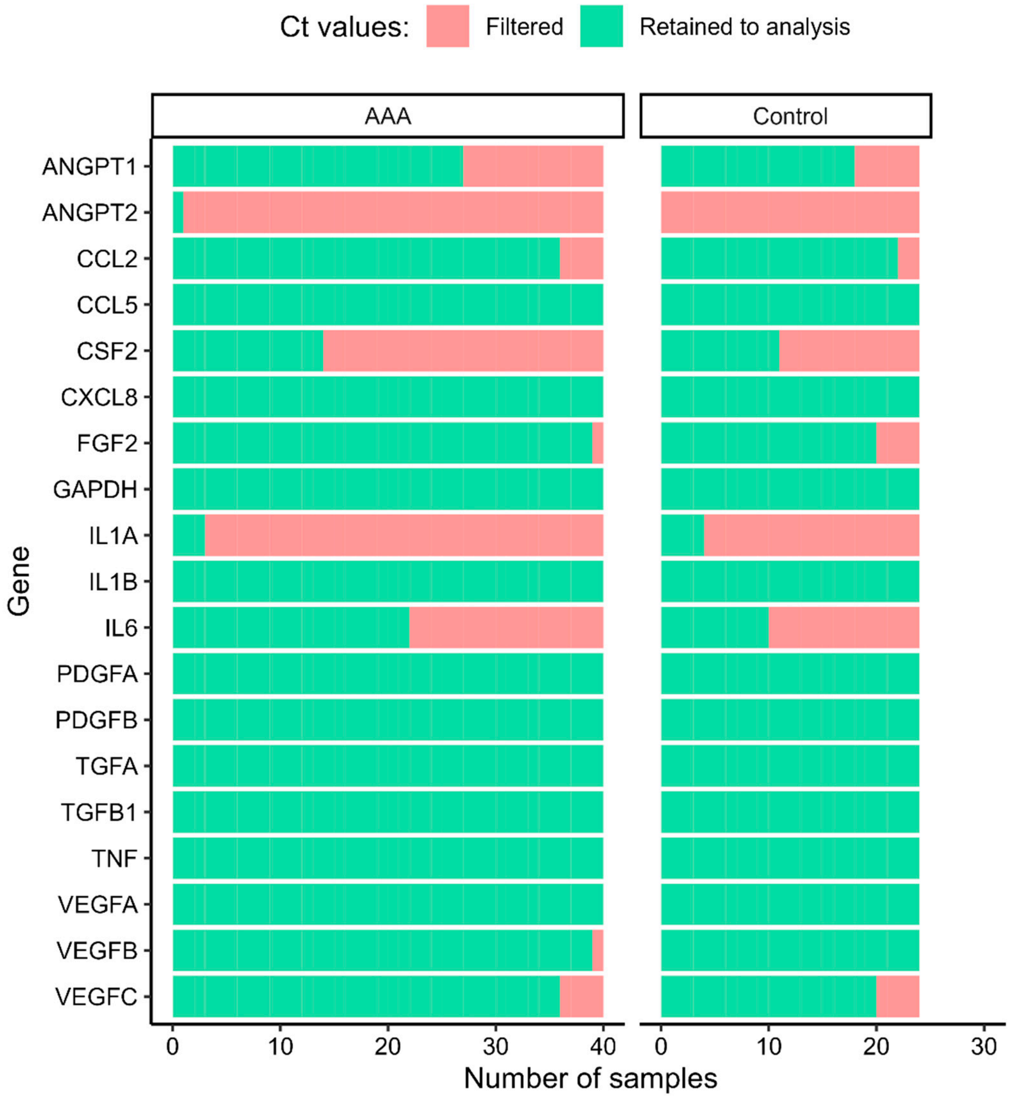

Figure S4. The amounts of samples with filtered and retained Ct values.

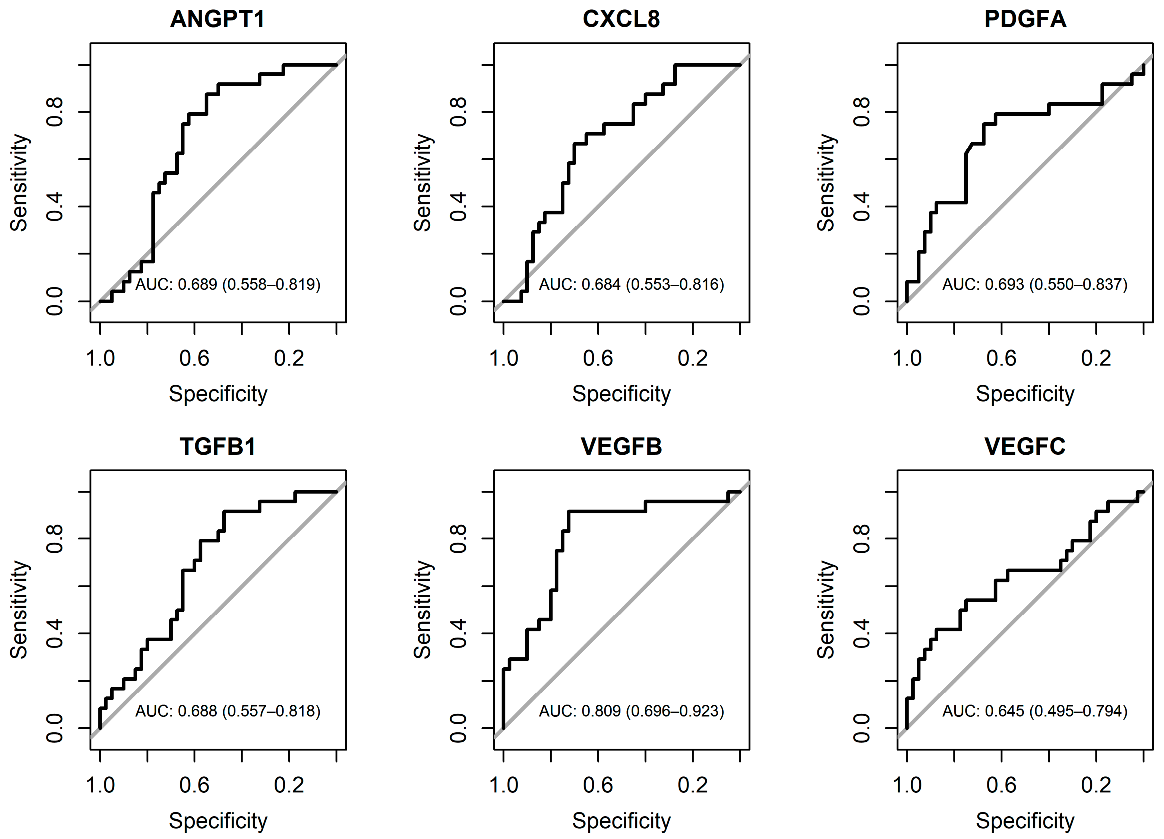

Figure S5. Results of Receiver Operating Characteristics (ROC) analysis performed for 6 genes selected from the comparison between AAA and control groups.

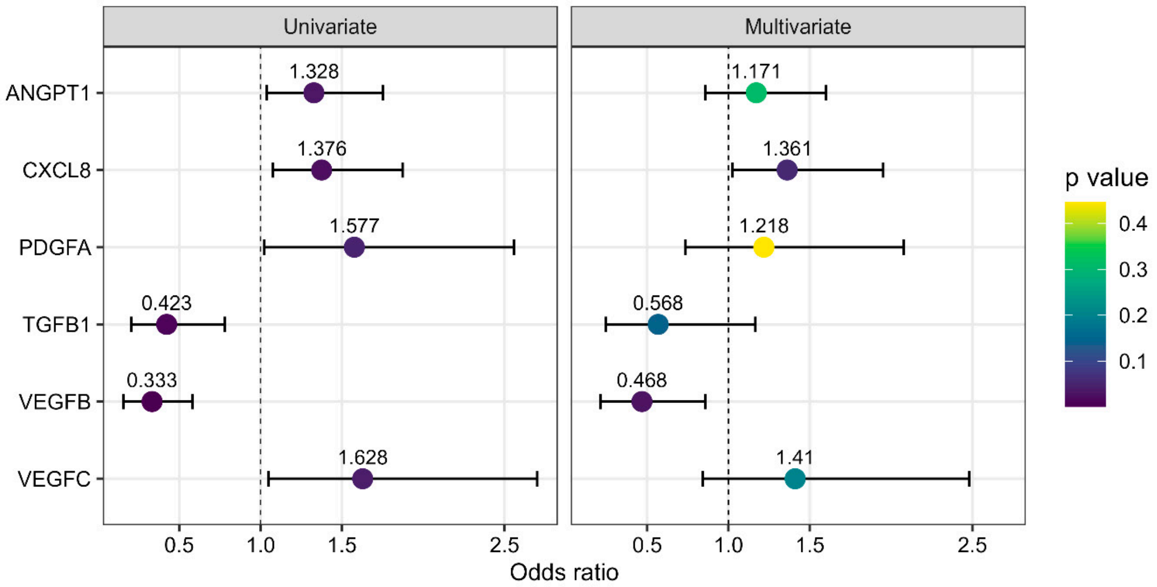

Figure S6. Odds ratios and their 95% confidence intervals and *p* values obtained in the univariate and multivariate logistic regression analysis (adjusted by age, sex, BMI, and smoking) for 6 genes selected from AAA vs. control comparison.

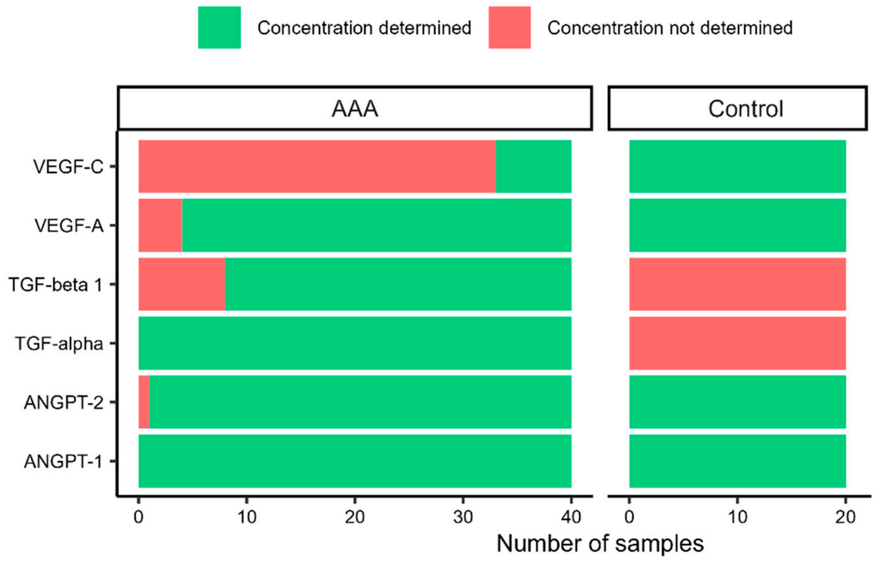

Figure S7. Amounts of samples in which plasma levels of analyzed proteins were determined or not.

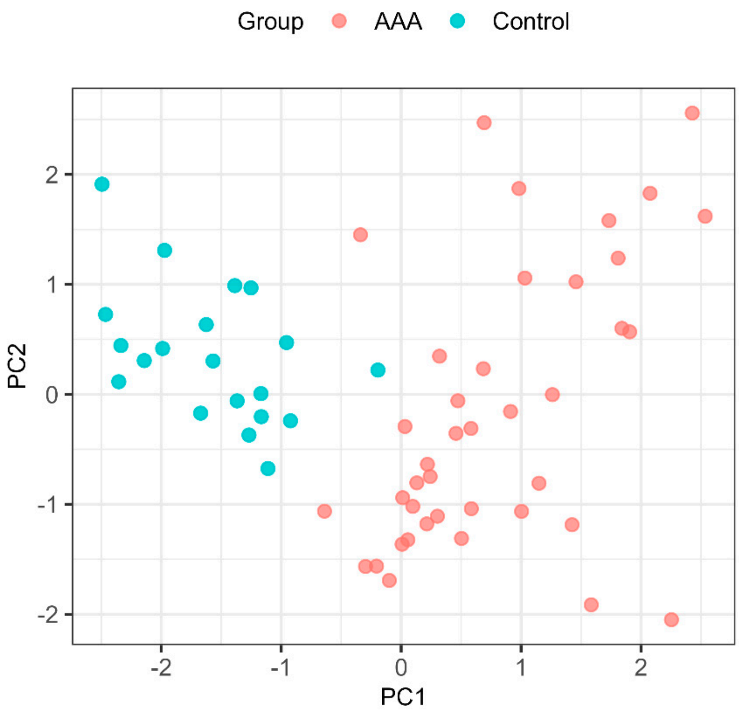

Figure S8. Spatial arrangement of samples using PCA components of protein plasma levels data.

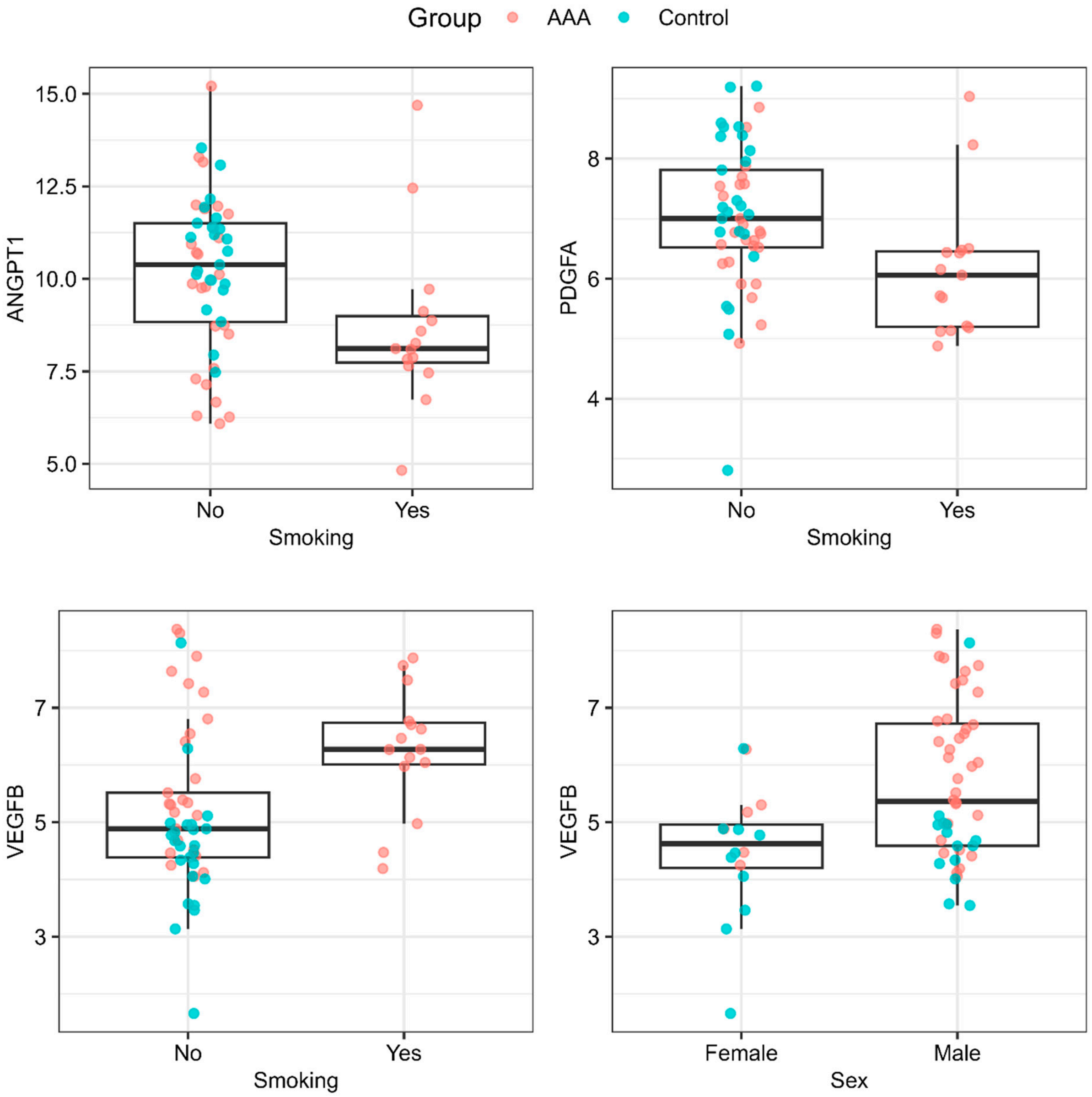

Figure S9. Expression of genes associated with sex and smoking status. Whiskers reach the most distant point in 1.5 interquartile range, boxes range between 25% and 75% quartile, horizontal lines inside boxes mark median value.

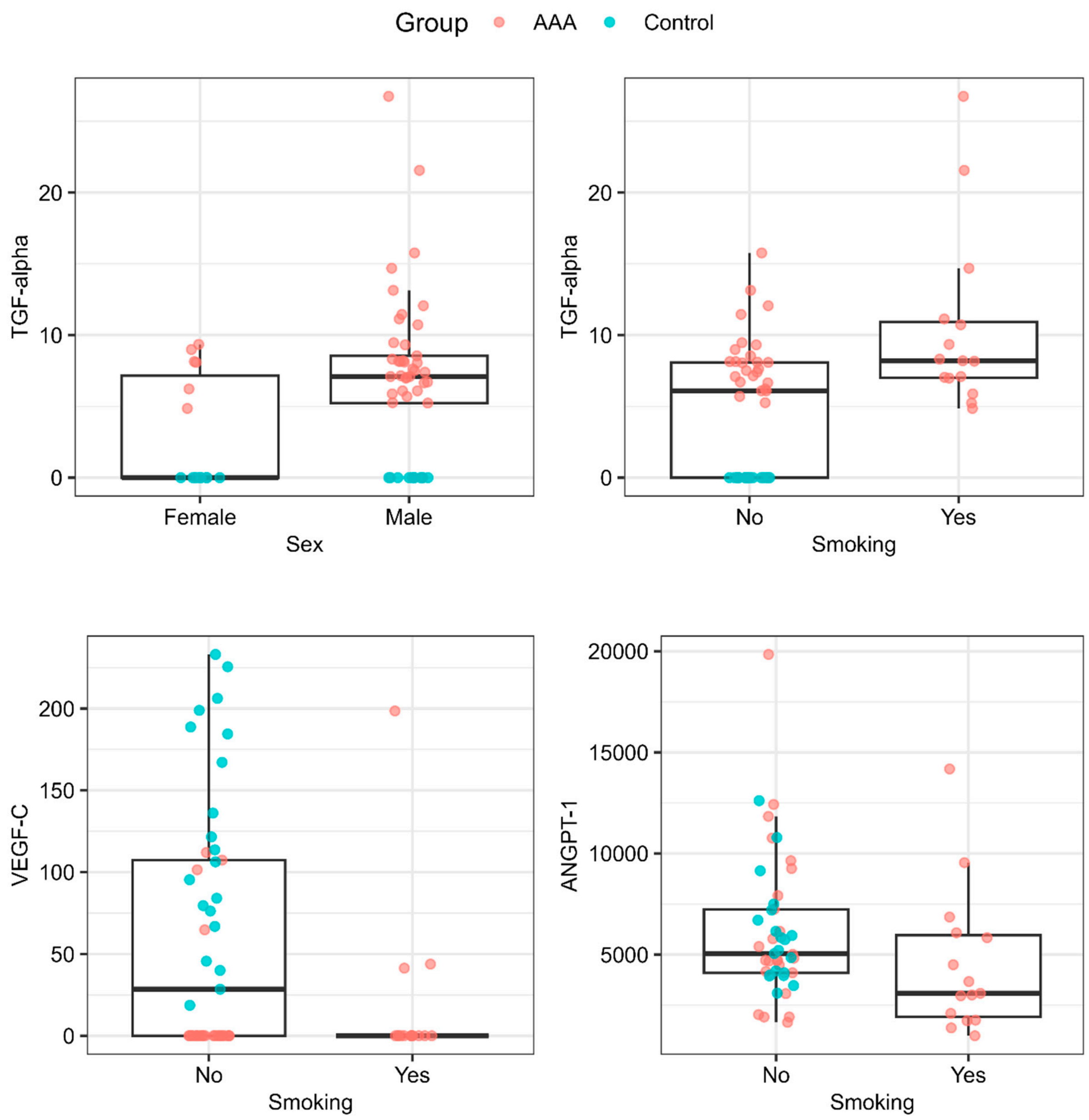

Figure S10. Plasma levels of proteins associated with sex and smoking status. Whiskers reach the most distant point in 1.5 interquartile range, boxes range between 25% and 75% quartile, horizontal lines inside boxes mark median value.

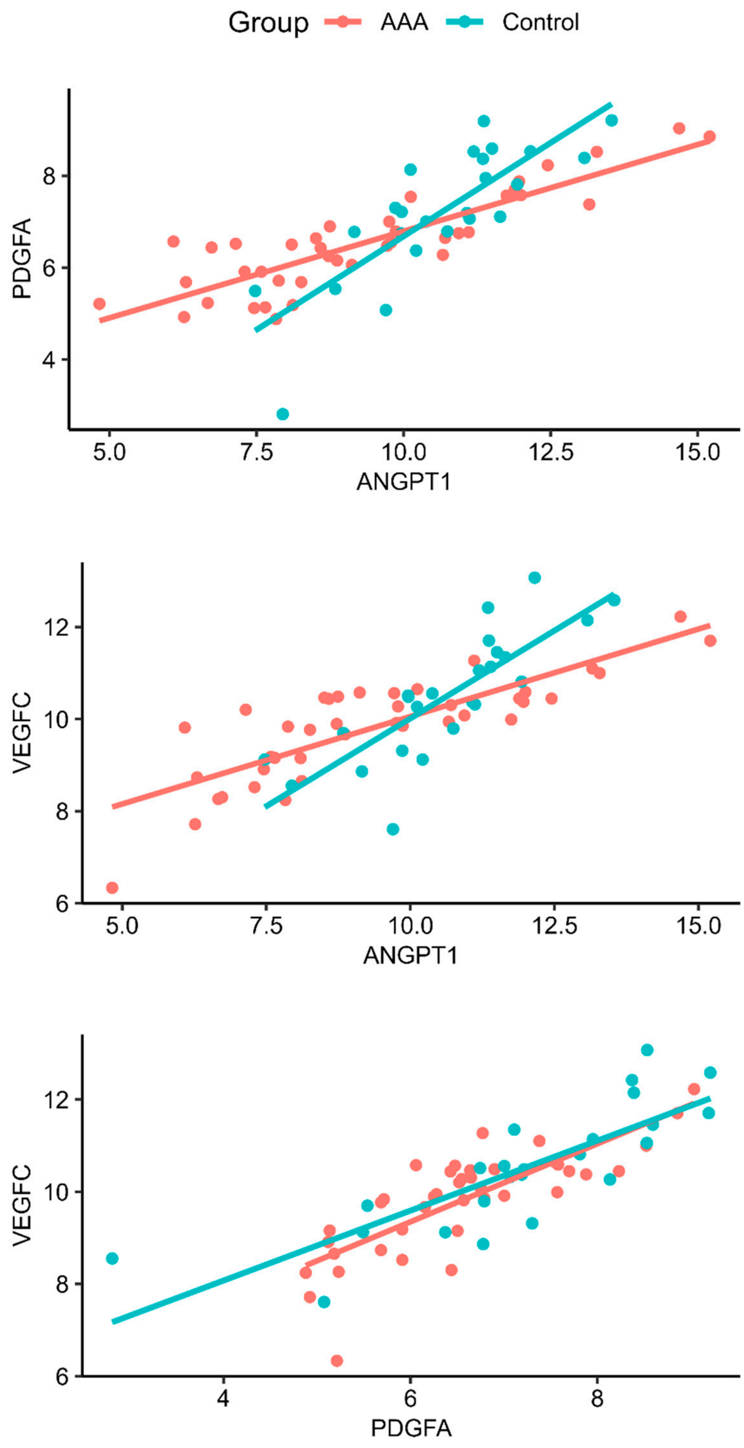

Figure S11. The strongest correlations found between 6 genes selected from the AAA vs. control comparison. Lines indicate the trend line (fitting line of the simple linear regression model).
